# Out-of-pocket health expenditure and associated factors among patients with hypertension in Debre-Tabor Comphrensive Specialized Hospital, South Gondar zone, Northwest Ethiopia, 2020

**DOI:** 10.3389/fpubh.2023.1014364

**Published:** 2023-05-04

**Authors:** Melkamu Alemayehu, Banchlay Addis, Tsega Hagos

**Affiliations:** ^1^Health Care Worker at Simada Primary Hospital, Gondar, Ethiopia; ^2^Department of Health Systems and Policy, College of Medicine and Health Science, University of Gondar, Gondar, Ethiopia

**Keywords:** out-of-pocket expenditure, expenditure, hypertension, catastrophic health care expenditure, Ethiopia

## Abstract

**Introduction:**

Hypertension is a non-communicable chronic disease that has a wide financial effect at the individual and household levels especially in developing countries due to its complexity and chronicity. Nevertheless, there are limited studies in Ethiopia. Therefore the aim of this study was to assess out-of pocket health expenditure and associated factors among adult patients with hypertension in Debre-Tabor Comphrensive Specialized Hospital.

**Methods:**

A facility-based cross-sectional study was conducted in total of 357 adult hypertensive patients from March to April 2020 using a systematic random sampling technique. Descriptive stastics were used to estimate the magnitude of out-of-pocket health expenditure, while after checking the assumptions linear regression model was fitted for identifying the factors associated with the outcome variable at a significance level of value of *p* < 0.05 and 95% confidence interval.

**Result:**

A total of 346 study participants interviewed with a response rate of 96.92%. Annual mean out of pocket health expenditure of the participant was $113.40 ± $10.18 with 95% CI = (102.63, 124.16) per patient. The direct medical mean out of pocket health expenditure of the participant was $68.86 per patient per year and the median of non-medical components of the out of pocket health expenditure of the participant was $3.53. Sex, wealth status, distance from hospital, comorbidity, health insurance and number of visit are factors significantly associated to out-of-pocket expenditure.

**Conclusion:**

This study revealed that out of pocket health expenditure among adult patients with hypertension was found high compared to the national *per capita* health expenditure. Sex, wealth index, distance away from hospital, frequency of visit, comorbidities, and health insurance coverage were factors significantly associated with high out-of-pocket health expenditure. Ministry of health together with regional health bureaus and other concerned stakeholders work on strengthening early detection and prevention strategies of chronic comorbidities of hypertensive patients,promote health insurance coverage and better to subsidize medication costs for the poors.

## Introduction

Hypertension is defined as a rise in systemic blood pressure above 140 mmHg or a rise in diastolic blood pressure above 90 mmHg (1). It is one of the most prevalent chronic noncommunicable diseases in the world, as well as a major risk factor for stroke, myocardial infarction, vascular disease, and chronic kidney disease (2, 3). Globally, an estimated 1.4 billion people had high blood pressure of 140/90 mmHg, accounting for 12.8% of all deaths and 18 million cardiovascular deaths each year, with two-thirds living in low-and middle-income countries (1, 3, 4). Hypertension is the leading cause of heart failure in Africa, where 46% of hypertensive adults develop cardiovascular disease (5). Hypertension prevalence in Ethiopia ranged from 12.5 to 28.37%, with a pooled prevalence of 19.6% (2, 5–8).

Out-of-pocket health expenditure (OOPHE) is defined as household spending incurred when using a service to obtain any type of health care (Promotive, preventive, curative, rehabilitative, palliative, or long-term) (9). According to the World Health Organization, catastrophic out-of-pocket health expenditure occurs when direct OOP payments exceed 40% of household income minus subsistence needs or OOP payments exceed 10% of total household income (10, 11). Household health expenditure is estimated to account for 45% of total global health expenditure and 23% of health expenditure in the developing world (12). An estimated 1.3 billion people worldwide lack access to effective and affordable health care. Approximately 170 million of those who do are forced to spend more than 40% of their household income on medical treatment (13). Every year, out-of-pocket health expenditure pushes 25 million households, or more than 100 million people, into poverty (14).

Between 2000 and 2015, out-of-pocket health spending contributed to global poverty and living below the poverty line (15). Financial risk-pooling mechanisms have been developed over several decades in developed countries’ health-care systems. Nonetheless, despite the existence of reasonably well-developed financial risk protection mechanisms, some households in these countries are still confronted with high out-of-pocket health expenditure (11).

In Ethiopia, poor health care financing remains a major challenge for the health system. In which the health system is dominated by low government spending, strong reliance on out-of-pocket health expenditure, inefficient and inequitable utilization of resources, poorly harmonized and unpredictable donor funding, households are vulnerable to impoverishment from out-of-pocket health expenditure (16). According to Ethiopia’s seventh national health accounts (2016), out-of-pocket health spending on general health was much higher than the global recommended target of 20% (17). However, much effort has been made to protect households from financial risks by increasing nominal and *per capita* health expenditures, establishing and expanding community-based health insurance (CBHI) schemes, and subsidizing some specific services (fee-waiver and exemption) (18, 19).

Hypertension is one of the 21st century’s silent killers and one of the most serious global public health issues, costing households more than other diseases.

In the United States, patients receiving hypertension treatment (13.1%) were significantly higher than other chronically ill (10.5%) and well patients (5.3%). Similarly, pooled data from the United States of America from 2003 to 2014 (20) revealed that the mean annual medical expenditure attributable to patients with hypertension was $9089 compared to patients without hypertension (21).

Direct OOP payments accounted for more than half of total health expenditures in the majority of low-income countries in 2007 (12). India is one of those countries that spends more than one-third of total income on cardiovascular diseases (CVD) and hypertension (22). The average annual cost of hypertension in Malawi was Rs. 5831.5 (23), while the average annual cost of hypertension in Kenya was $475 (24).

According to some studies on the cost of hypertension conducted in Ethiopia, the mean annual cost of hypertension is high (ranging from $91.72 to US $267.6) (25, 26). According to an Addis study on cardiovascular disease, approximately 27% of households (26.7, 95% CI 23.1 to 30.6) had catastrophic health expenditure (10).

According to studies conducted on hypertension out-of-pocket expenditure in Columbia and Malawi, the average annual hypertension-attributable out-of-pocket expenditure was USD $147.75 and Rs. 4042 and Rs. 7621 for government and private facilities, respectively (23, 27). Findings in Pakistan revealed that, in addition to higher OOP medical spending, the incidence of catastrophic health expenditure is higher for BPD (blood pressure and diabetes) medication consuming households, reaching as high as 12.96% among BPD medication consuming households and only 5.84% among households “not” consuming BPD medication (20). Furthermore, a study on the OOP of HTN conducted in Addis Abeba revealed that the total out-of-pocket expenditure was estimated to be 5279.50 (mean 7194.00) birr/month (28).

Out-of-pocket health expenditure is influenced by a variety of factors, which are broadly classified as socioeconomic, clinical, and payment type. In the literature, socio-demographic and economic factors such as occupation (29), education, family size, sex, age, marital status (30), residence, and wealth index (29) were factors influencing out-of-pocket expenditure. Payment type factors include drug cost (29), health insurance membership (21, 29), waived or exempted service users, and distance from the hospital (26). Clinical factors include the presence of complications, the duration of the disease, and the stage of hypertension (30).

In general, hypertension is a major contributory factor to subsequent morbidity and mortality in Ethiopia, and out-of-pocket health spending for hypertension imposes a significant economic burden on patients and societies. As the health system is dominated by out-of-pocket payment systems, which push individuals and households into poverty and disrupt household welfare. There have been few studies on the magnitude of hypertensive patients’ out-of-pocket health expenditure,which didnot include the detailed factors and the study time is some what long. As a result, the purpose of this study is to estimate the burden of out-of-pocket health expenditure and identify the associated factors in hypertensive patients, which helps to provide updated baseline information for health planners on how to intervene.

## Methods

### Study design, period and setting

This facility-based cross-sectional study was conducted at Debre-Tabor Referral Hospital Chronic Illness Follow-up Outpatient department (OPD) from March to April 2020 to assess the level of out-of-pocket health expenditure among patients with hypertension.

From March to April 2020, the Debre-Tabor Comphrensive Specialized Hospital Chronic Illness Follow-up Outpatient Department (OPD) conducted a facility-based cross-sectional study to assess the level of out-of-pocket health expenditure among hypertensive patients.

Debre-Tabor Comprhensive Specialized Hospital is located in South Gondar Administrative Zone, Amhara National Regional State, 667 kilometers from Addis Ababa (Ethiopia’s capital city) and 97 kilometers from Bahir Dar (the regional capital). Currently, the hospital is one of Ethiopia’s and the Amhara region’s Comprhensive Specialized health care facilities, serving 2,609,823 people in the South Gondar zone and the surrounding zones. It offers an outpatient hypertensive clinic 5 days a week, with a patient flow of approximately 721 hypertensive patients per month.

### Sample size determination

The sample size for the first objective is calculated using a single population mean formula from a previous study done in Ethiopia in public hospitals at Addis Abeba (13). The sample size was 357 based on the assumptions Zà/2 1.96, and annual mean ± SDof out-of-pocket health expenditure with of $158.64 ± $29, and a margin of error (d) of 2% of the mean. On average, every month, around 721 patients come to the hypertensive outpatient clinic. Using systematic random sampling with a *K* value of 2 (721/357), every other hypertensive patient was selected and interviewed for the study.

The population consisted of all adult hypertensive patients attending an outpatient chronic illness follow-up clinic in Debre-Tabor Comprhensive and Speciaalized Hospital during the study. All patients who were diagnosed with hypertension for at least 1 year were included, while patients who were unable to communicate were excluded.

### Data collection tools and procedure

A Semi-structured interviewer administered questionnaire adopted by reviewing different literature was used. The tool comprises an out-of-pocket health expenditure questionnaire with 16 items, socio-economic and demographic characteristics, clinical, and payment type/modalities to collect information from the patients. From March 15 to April 20, 2020, three diploma nurses with one supervisor were assigned for data collection. The questionnaire was initially prepared in English and translated into Amharic (the local language) for understandability, and after data collection, it was retranslated back to English to keep its consistency.

Direct medical payments are defined as payments directly related to the illness or condition, such as medication, folder fees, consultation, and laboratory test expenses for hypertensive patients, and are measured as values per visit per patient per month in ETB.

*Direct non-medical payments* are defined as payments which are not directly related to the illness or condition but indirectly related to the illness, which includes transport and food for hypertension patients and their caregivers’ expenditure measured per visit per month.

*Indirect payment* was a time devoted by hypertensive patients and their caregivers to seeking treatment for 1 year.

*Out-of-pocket health expenditure is referred* to as payments (OOPs) directly or indirectly related to the illness or condition, which includes direct medical payments and non-medical payments. Which were measured by out-of-pocket health expenditure per visit per patient per month, after which the expenditure changed to annual mean values per patient. Finally, the result of the study was reported. According to the commercial bank of Ethiopia, the annual exchange rate between the USD and the Ethiopian Birr (ETB) on May 25, 2020 was USD 1.0 = ETB 33.99.

### Data quality control measures

To ensure the quality of the data, one-day training on the contents of the questionnaire and the purpose of the study was given to the data collectors and supervisor. A pre-test was done 1 week before the actual data collection among 18 patients in Wogeda Primary Hospital. During the pretest, the questionnaire was checked for its clarity, simplicity, understandability, completeness, consistency, and coherency. Quality of data was also controlled through conducting close supervision and follow-up by supervisors and principal investigators on the filled questionnaire.

### Data management and analysis

Before being exported to Stata14 software for further analysis, all data was entered into Epidata version 4.6, coded, cleaned, and checked for completeness. The wealth index was classified into five quintiles using principal component analysis, which was then analyzed using state 14 software. Summary statistics for the outcome and independent variables were computed. After checking linear regression assumptions such as linearity, normality, homoscedasticity, equal variance, and multicollinearity, simple linear regression and multiple linear regression were used to identify factors associated with out-of-pocket health expenditure. Variables with a *p*-value <0.05 at 95% confidence intervals were considered statistically significant in multiple linear regression analysis.

## Result

### Socio-economic and demographic factors

A total of 346 hypertensive patients participated in the study, with a response rate of 96.92%. The majority of the participants, 212 (61.27%) were city dwellers. The majority 220 (63.58%) of the participants were aged between 44 and 64 years, and the mean age of the participants was 54.25 ± 11.02. Among the total participants, 70 (20.24%) were the poorest. The majority of participants, 198 (57.23%), travel less than five kilometers to the health facility (Table 1).

**Table 1:** Socio-demographic and economic characteristics of study participants at Debre-Tabor General Hospital, North West Ethiopia, 2020 (*n* = 346).

Variable	Frequency	Percent (%)	Mean ± SD
Residence			
Urban	212	61.27	
Rural	134	38.73	
Sex			
Male	225	65.03	
Female	121	34.97	
Age (in years)			54.25 ± 11.02
18–44	58	16.76	
44–64	220	63.58	
≥64	68	19.66	
Marital status			
Single	12	3.47	
Married	266	76.88	
Divorced	19	5.49	
Windowed	48	13.87	
Separated	1	0.29	
Religion			
Orthodox Christian	270	78.03	
Muslim	66	19.08	
Protestant	10	2.89	
Educational status			
Unable to read and write	123	35.55	
Able to read and write only	68	19.65	
Grade 1–8	30	8.67	
Grade 9–12	30	8.67	
College and above	95	27.46	
Occupational status			
Farmer	106	30.64	
House wife	42	12.14	
Employed	97	28.03	
Merchant	55	15.90	
Daily Labor	15	4.34	
Others	31	8.96	
Wealth status			
Poorest	70	20.24	
Poor	69	19.94	
Middle	69	19.94	
Rich	69	19.94	
Richest	69	19.94	
Distance from the health facility			4
Less than 5 km	198	57.23	
5 km-10 km	35	10.12	
≥10 km	113	32.66	
Means of transport			
Foot	190	54.91	
Vehicle	156	45.09	

### Clinical related factors and payment type factors

The median duration of the diseases was 4 years. The majority of participants (340, or 98%) were followed once per month. While 88 (25.43%) of participants had comorbidities, of which 47 (53.41%) had diabetes mellitus. Furthermore, 86 (24.85%) of the participants had health insurance. The majority of the participants’ expenses (258, or 74.57%) were covered by cash, with 194 (56.23%) covered by their own savings (Table 2).

**Table 2:** Clinical related factors of study participants at Debre-Tabor General Hospital, North west Ethiopia, 2020 (*n* = 346).

Variables	Frequency	Percent (%)	Median
Duration of the diseases(in years)			
<5 years	255	73.70	
5–10 years	76	21.97	4
≥10 years	15	4.33	
Frequency of visit			
Less than 2 times	340	98.27	
≥2 times	6	1.73	
Comorbidities			
Yes	88	25.43	
No	258	74.57	
Types of comorbidities			
Diabetes mellitus	47	53.41	
Kidney diseases	7	7.95	
Cardiac disease	23	26.14	
Epilepsy	2	2.27	
Asthma	9	10.23	

### Out of pocket health expenditure

The participant’s annual mean out-of-pocket health expenditure was $113.40 ± $10.18 with a 95% CI = (102.63, 124.16) per patient. The direct medical components of the participant’s mean out-of-pocket health expenditure were $68.86, 95% CI = (61.65, 76.07) per patient per year. The non-medical components of the participant’s median out-of-pocket health expenditure were $3.53 [95% CI = (13.04, 18.40)] per patient per year.

### Factors associated with out of pocket health expenditure

Multiple linear regression analysis was conducted after checking assumptions in the final model. The following bivariable regression variables were chosen for the final model multiple linear regression analysis with *p*-value <0.2: gender, marital status, religion, wealth index, distance from hospital, frequency of visit, comorbidity, and health insurance coverage.

Being a female participant expended $17.45 unit more [95% CI = (0.81, 34.09)] than male participants. When comparing out of pocket health expenditure the richest to the poorest, the richest expend extra $31.02 [95% CI = (6.54, 55.50)].

Out-of-pocket health expenditure among hypertensive patients who traveled more than 10 kilometers from the hospital is 71.45 dollars more compared to patients who traveled less than 5 kilometers.

A participant with comorbidities had an increased out-of-pocket health expenditure of $53.66 (95% CI = 34.55, 72.77) as compared with patients without comorbidities.

Out-of-pocket health expenditure for those havenot health insurance is $98.89 more; 95% CI = (79.36, 118.42) compared to those who had health insurance coverage. Participants who had 2 or more visits had an increased out-of-pocket health expenditure of $374.09 [95% CI = (315.35, 432.84)] as compared to those participants who had one visit (Table 3).

**Table 3:** Factors associated with out of pocket health expenditure among patients with hypertension in Debre-Tabor General Hospital, Northwest Ethiopia, 2020.

Variables	COR	*p*-value	AOR	*p*-value	(95% CI)
Sex					
Male	Ref	–	Ref	–	–
Female	21.47	0.062	17.45	0.04	(0.81,34.09)*
Maritial status					
Single	Ref	–	Ref	–	–
Married	−91.21	0.002	−40.64	0.06	(−82.74, 1.46)
Divorced	−78.23	0.033	−33.99	0.20	(−86.17,18.18)
Widowed	−34.23	0.285	−13.97	0.55	(−59.80,31.85)
Separated	−133.6	0.197	−76.86	0.33	(−231.05,77.32)
Religion					
Orthodox	Ref	–	Ref	–	–
Muslim	−19.9	0.155	−12.89	0.20	(−32.43,6.64)
Protestant	−22.2	0.49	−0.56	0.98	(−48.96,47.84)
Wealth Status					
Poorest	Ref	–	Ref	–	–
Poor	−3.21	0.85	5.59	0.69	(−21.59,32.77) 32.77111
Medium	10.04	0.55	14.80	0.29	(−12.47,42.07)
Rich	7.6	0.65	4.80	0.70	(−19.41,29.10)
Richest	56.8	0.001	31.02	0.01	(6.54, 55.50)*
Distance from Hospial (km)					
<5	Ref	Ref	Ref	–	–
5–10	4.22	0.82	20.85	0.15	(−7.87,49.56)
≥10	29.4	0.014	71.45	0.000	(51.56, 91.34)**
Frequency of visit (wk)					
<2visit	Ref		Ref	–	–
≥2 visit	392.1	0.001	374.09	0.000	(315.35,432.84)**
Comorbidities					
No	Ref	–	Ref	–	–
Yes	83.8	0.001	53.66	0.000	(34.55,72.77)**
Health Insurance					
Yes	Ref	–	Ref	–	–
No	61.55	0.001	98.89	0.000	(79.36,118.42)**

*Significant at *p*-value ≤0.05. **Significant at *p*-value ≤0.001. Km = kilometer.

## Discussion

Hypertension is a common chronic public health problem having a profound effect on individual and household wealth, especially for out-of-pocket payers. The aim of this study was to determine the out-of-pocket expenditure of hypertension patients and associated factors.

This study revealed that the mean annual out-of-pocket health expenditure of patients with hypertension was $113.4 ± $10.18; 95% CI = (102.63, 124.16)/patient/year. This finding is higher than the 7^th^ national health account data in 2016/2017, *per capita*l health expenditure report and study conducted at the University of Gondar Comprehensive Specialized Hospital Northwest Ethiopia ($33.2) and ($v91.72) respectively (17, 25). The possible explanation for this variation might be the study period, the status of the diseases in the participant, or distance from the health facility. On the other hand, it was lower than the studies conducted in Addis Ababa (10). The possible explanation for this variation might be that the study setting difference, Addis abeba is the large city and debre-tabor is zone capital city the expenditure for transport and other activities is different, which leads to an increase in their expenditure. The findings of this study were also lower than those of previous studies conducted in Kenya (24). This disparity could be explained by differences in study settings, time periods, expenditure estimation methods, and participant socioeconomic status. This finding is also lower than that of previous studies in Ethiopia’s Southwest Shewa Zone, Oromia Regional State (26). This difference may be due to the fact that a study conducted in Southwest Shewa estimates both direct and indirect costs of hypertension, whereas this study does not.

Female hypertensive patients spend $17.45 more per day than male hypertensive patients [95% CI = (0.81, 34.09] This could be explained by the fact that females are more likely to have supporters and use vehicles for transportation than males, causing them to spend more. A study conducted in the United States (31) lends support to this study.

When compared to the poorest, out-of-pocket health expenditure among the richest is $31.02 more [95% CI = (6.54, 55.50)].The possible explanation might be that the richest have a high income and their wants also vary a lot compared to the poorest, who have limited wants and expend less. This study is supported by studies conducted in Ethiopia, Kenya, Namibia, Nigeria, Albania, Bangladesh, and India which found that the richest individuals and households had higher out-of-pocket expenditures on health care than the poorest households (24, 31–36). Those who belong to the highest Socio-economic Status have a better capacity to pay for health care services, expensive drugs, and diagnosis modalities.

In this study, hypertensive patients who lived 10 kilometers or more from the hospital spent $71.45 more [95% CI = (51.56, 91.34)] than hypertensive patients who lived less than 5 kilometers from the hospital. It is supported by studies conducted in Ethiopia (37). The possible explanation could be that hypertensive patients who live far from health care facilities spend more money on transportation and food than those who live close to the hospital.

Participants who visited the hospital twice or more spent an additional $374.09 [95% CI = (315.35, 432.84)] than participants who visited the hospital once. This disparity may be explained by the fact that those who visit the hospital frequently spend more money on transportation, food, and drugs than those who visit the hospital less frequently. Which is not supported by previous studies.

Hypertensive patients with comorbidities spent $53.66 more [95% CI = (34.55, 72.77)] than those without comorbidities. This was supported by a study conducted at the University of Gondar Comprehensive Specialized Hospital Northwest Ethiopia and the US (25, 31). This could be explained by the fact that participants with comorbidities may have spent more money on extra drugs and laboratory tests.

This study also revealed that health insurance coverage has a significant association with out-of-pocket health expenditure. Patients with hypertension who lacked health insurance spent $98.89 [95% CI = (79.36, 118.42)] more than those who did have health insurance. This study was supported by studies conducted in Kenya, Korea, and the US (24, 31, 38). The possible explanation might be that those participants who do not have health insurance may delay care due to financial problems, which leads to complications that demand extra money for different aspects, where as having health insurance membership enables the members to get a service at health facilities free of medication and service charge at the time of hypertension follow up.

The information required in calculating costs was based on an investigation of patients and their households rather than documenting.

This study tries to identify factors influencing out-of-pocket expenditure beyond determining out-of-pocket expenditure might be taken as a strength of the study. On the contrary, the limitations are that, as the study includes only adult hypertensive patients attaining outpatient department, this finding may not be generalizable for those patients in inpatient department.

Despite using different leading techniques to minimize recall bias, remembering each cost they expend on each activity is difficult, so there might be underestimation or overestimation.

## Conclusion

This study revealed that out-of-pocket health expenditure among adult patients with hypertension was found to be high compared to the national per capita health expenditure. Sex, wealth index, distance from hospital, frequency of visit, comorbidities, and health insurance coverage were associated with high out-of-pocket health spending.

The Ministry of health should provide a better subsidy on the service and medication fees for hypertensive patients. And MOH together with regional health bureaus and other concerned stakeholders, working on strengthening early detection and prevention strategies for chronic comorbidities in hypertensive patients. In addition, the Ethiopian health insurance agency needs to strengthen efforts in implementing strategies that accelerate the enrollment of the community into health insurance schemes, expand social health insurance schemes, and expend other coping mechanisms. Furthermore, healthcare providers focus on primordial prevention to minimize the severity of hypertension diseases and comorbidities and provide better care at home for hypertensive patients to reduce transportation, expenditure, and comorbidities in collaboration with health posts and community health workers. Conducting longitudinal and community-based research on out-of-pocket health expenditure among hypertensive patients is better for more generalization.

## Data availability statement

The raw data supporting the conclusions of this article will be made available by the authors, without undue reservation.

## Ethics statement

The studies involving human participants were reviewed and approved by Ethical review board of University of Gondar. The patients/participants provided their written informed consent to participate in this study. Written informed consent was obtained from the individual (s) for the publication of any potentially identifiable images or data included in this article.

## Author contributions

MA and BA conceptualized the study, analyzed the data, and prepared the manuscript. TH analyzed and helped with writing the manuscript. BA developed and provided feedback for all sections of the review protocol and approved the final manuscript. All authors have read and approved the final manuscript.

## Funding

This is part of a master thesis funded by University of Gondar. The funders had no role in the study design, data collection, analysis, decision to publish, or preparation of the manuscript.

## Conflict of interest

The authors declare that the research was conducted in the absence of any commercial or financial relationships that could be construed as a potential conflict of interest.

The reviewer TD declared a shared affiliation with the authors to the handling editor at the time of review.

## Publisher’s note

All claims expressed in this article are solely those of the authors and do not necessarily represent those of their affiliated organizations, or those of the publisher, the editors and the reviewers. Any product that may be evaluated in this article, or claim that may be made by its manufacturer, is not guaranteed or endorsed by the publisher.

## Abbreviations

BPD: Blood pressure and diabetes
CBHI: Community Based Health Insurance
CVD: Cardio Vascular Disease
HTN: Hypertension
OOP: Out of Pocket
OOPHE: Out of Pocket Health Expenditure
USD –PPP: Purchasing Power Party
WHO: World Health Organization

## References

[1] Forouzanfar MH Liu P Roth GA Ng M Biryukov S Marczak L. Global burden of hypertension and systolic blood pressure of at least 110 to 115 mm Hg, 1990–2015. JAMA. (2017) 317:165–82. doi: 10.1001/jama.2016.19043, PMID: 28097354

[2] Belachew A Tewabe T Miskir Y Melese E Wubet E Alemu S. Prevalence and associated factors of hypertension among adult patients in Felege-Hiwot comprehensive referral hospitals, northwest, Ethiopia: a cross-sectional study. BMC Res Notes. (2018) 11:1–6. 30526686 10.1186/s13104-018-3986-1 PMC6288930

[3] World Health Organization. Clinical guidelines for the management of hypertension. Geneva: World Health Organization (2005).

[4] Unger T Borghi C Charchar F Khan NA Poulter NR Prabhakaran D. 2020 International Society of Hypertension global hypertension practice guidelines. Hypertension. (2020) 75:1334–57. doi: 10.1161/HYPERTENSIONAHA.120.15026, PMID: 32370572

[5] Kiber M Wube M Temesgen H Woyraw W Belay YA. Prevalence of hypertension and its associated factors among adults in Debre Markos town, Northwest Ethiopia: community based cross-sectional study. BMC Res Notes. (2019) 12:1–6. 31307528 10.1186/s13104-019-4431-9 PMC6631738

[6] Abebe SM Berhane Y Worku A Getachew A. Prevalence and associated factors of hypertension: a crossectional community based study in Northwest Ethiopia. PLoS One. (2015) 10:e0125210. doi: 10.1371/journal.pone.0125210, PMID: 25909382 PMC4409323

[7] Asresahegn H Tadesse F Beyene E. Prevalence and associated factors of hypertension among adults in Ethiopia: a community based cross-sectional study. BMC Res Notes. (2017) 10:1–8. doi: 10.1186/s13104-017-2966-1 29183367 PMC5704552

[8] Chuka A Gutema BT Ayele G Megersa ND Melketsedik ZA Zewdie TH. Prevalence of hypertension and associated factors among adult residents in Arba Minch Health and demographic surveillance site, southern Ethiopia. PLoS One. (2020) 15:e0237333. doi: 10.1371/journal.pone.0237333, PMID: 32776993 PMC7416932

[9] World Health Organization. WHO malaria report 2018. Geneva: World Health Organization (2018).

[10] Tolla MT Norheim OF Verguet S Bekele A Amenu K Abdisa SG. Out-of-pocket expenditures for prevention and treatment of cardiovascular disease in general and specialised cardiac hospitals in Addis Ababa, Ethiopia: a cross-sectional cohort study. BMJ Glob Health. (2017) 2:e000280. doi: 10.1136/bmjgh-2016-000280, PMID: 29242752 PMC5584490

[11] Xu K Evans DB Kawabata K Zeramdini R Klavus J Murray CJL. Household catastrophic health expenditure: a multicountry analysis. Lancet. (2003) 362:111–7. doi: 10.1016/S0140-6736(03)13861-5, PMID: 12867110

[12] Headley Jennifer B.J.N., Measuring houshold outof pockect expenditure, consideration of social enterprise and organizations in low and middile income countries (2016).

[13] Xu K Evans DB Kawabata K Zeramdini R Klavus J Murray CJL. Household catastrophic health expenditure: a multicountry analysis. Lancet. (2003) 362:111–7. doi: 10.1016/S0140-6736(03)13861-5, PMID: 12867110

[14] World Health Organization. Designing health financing systems to reduce catastrophic health expenditure. Geneva: World Health Organization (2005).

[15] World Health Organization. Global monitoring report on financial protection in health 2019. Geneva: World Health Organization (2020).

[16] EPHI, Improving health care financing in Ethiopia. (2014).

[17] Democratic F. Ethiopia R.O. Ministry of Health, Ethiopian health accounts household Health service utilization and expenditure survey 2015/16 (2017).

[18] Democratic F Ethiopia Ro Ministry of Health. Ethiopia’s Fifth National Health Accounts, 2010/2011 April 2014. Addis: Ababa.

[19] EPHI, Improving Health care financing in Ethiopia report what is a policy dialogue? Who participated in the dialogue? (2014).

[20] Datta BK Husain MJ Asma S. Assessing the relationship between out-of-pocket spending on blood pressure and diabetes medication and household catastrophic health expenditure: evidence from Pakistan. Int J Equity Health. (2019) 18:1–12. doi: 10.1186/s12939-018-0906-x 30646905 PMC6334430

[21] Kirkland EB Heincelman M Bishu KG Schumann SO Schreiner A Axon RN. Trends in healthcare expenditures among US adults with hypertension: national estimates, 2003–2014. J Am Heart Assoc. (2018) 7:e008731. doi: 10.1161/JAHA.118.008731, PMID: 29848493 PMC6015342

[22] Veisani Y Jenabi E Nematollahi S Delpisheh A Khazaei S. The role of socio-economic inequality in the prevalence of hypertension in adults. J cardiovasc Thorac Res. (2019) 11:116–20. doi: 10.15171/jcvtr.2019.20, PMID: 31384405 PMC6669432

[23] Thakare BS Adhav A Kadam S. Economic burden of hypertension care on households of Malwani slum of Mumbai: a cross-sectional study. Int J Res Med Sci. (2015) 3:2376.

[24] Oyando R Njoroge M Nguhiu P Kirui F Mbui J Sigilai A. Patient costs of hypertension care in public health care facilities in Kenya. Int J Health Plann Manag. (2019) 34:e1166–78. doi: 10.1002/hpm.2752, PMID: 30762904 PMC6618067

[25] Adane E Atnafu A Aschalew AY. The cost of illness of hypertension and associated factors at the University of Gondar Comprehensive Specialized Hospital Northwest Ethiopia, 2018. Clin Econom Outcomes Res. (2020) 12:133–40. doi: 10.2147/CEOR.S234674, PMID: 32184636 PMC7064277

[26] Zawudie AB Lemma TD Daka DW. Cost of hypertension illness and associated factors among patients attending hospitals in Southwest Shewa Zone, Oromia Regional State, Ethiopia. ClinicoEconomics Outcomes Res. (2020) 12:201. 10.2147/CEOR.S241591 PMC7154006 32308448

[27] Londoño Agudelo E García Fariñas A Pérez Ospina V Taborda Pérez C Villacrés Landeta T Battaglioli T. Out-of-pocket expenditure for hypertension care: a population-based study in low-income urban Medellin, Colombia. Glob Health Action. (2020) 13:1806527. doi: 10.1080/16549716.2020.1806527, PMID: 32867605 PMC7480425

[28] Bedane SN. Out of pocket expenditures among hypertensive patients and their households who visit public hospitals in Addis Ababa, Ethiopia, 2016. Health Econ Outcome Res. (2018) 4:1–7.

[29] Okello NO Njeru A. Factors affecting out-of-pocket medical expenditure among out patients in hospitals in Nairobi county. People. (2013)

[30] Zhang X Xu Q Guo X Jing Z Sun L Li J. Catastrophic health expenditure: a comparative study between hypertensive patients with and without complication in rural Shandong, China. BMC Public Health. (2020) 20:545. doi: 10.1186/s12889-020-08662-0, PMID: 32321485 PMC7178564

[31] Bernard DM Johansson P Fang Z. Out-of-pocket healthcare expenditure burdens among nonelderly adults with hypertension. Am J Manag Care. (2014) 20:406–13. 25181569

[32] Gustafsson-Wright E Janssens W Van Der Gaag J. The inequitable impact of health shocks on the uninsured in Namibia. Health Policy Plan. (2011) 26:142–56. doi: 10.1093/heapol/czq029, PMID: 20668002

[33] Hotchkiss DR Hutchinson PL Malaj A Berruti AA. Out-of-pocket payments and utilization of health care services in Albania: evidence from three districts. Health Policy. (2005) 75:18–39. doi: 10.1016/j.healthpol.2005.02.003, PMID: 16298226

[34] Onwujekwe O Hanson K Ichoku H Uzochukwu B. Financing incidence analysis of household out-of-pocket spending for healthcare: getting more health for money in Nigeria? Int J Health Plann Manag. (2014) 29:e174–85. doi: 10.1002/hpm.2166, PMID: 23390079

[35] Rahman MM Gilmour S Saito E Sultana P Shibuya K. Health-related financial catastrophe, inequality and chronic illness in Bangladesh. PLoS One. (2013) 8:e56873. doi: 10.1371/journal.pone.0056873, PMID: 23451102 PMC3581555

[36] Prinja S Jagnoor J Sharma D Aggarwal S Katoch S Lakshmi PVM. Out-of-pocket expenditure and catastrophic health expenditure for hospitalization due to injuries in public sector hospitals in North India. PLoS One. (2019) 14:e0224721. doi: 10.1371/journal.pone.0224721, PMID: 31697781 PMC6837486

[37] Bogale AB Lemma TD Daka DW. Cost of hypertension illness and associated factors among patients attending hospitals in Southwest Shewa Zone, Oromia Regional State, Ethiopia. Clinicoecon Outcomes Res. (2019) 12:201–11. doi: 10.2147/CEOR.S241591 PMC7154006 32308448

[38] Lee W-Y Shaw I. The impact of out-of-pocket payments on health care inequity: the case of national health insurance in South Korea. Int J Environ Res Public Health. (2014) 11:7304–18. doi: 10.3390/ijerph110707304, PMID: 25046630 PMC4113877

